# Assessment of coverage of preventive treatment and insecticide-treated mosquito nets in pregnant women attending antenatal care services in 11 districts in Mozambique in 2011: the critical role of supply chain

**DOI:** 10.1186/s12936-017-1872-2

**Published:** 2017-05-25

**Authors:** Cristolde Salomão, Jahit Sacarlal, Eduardo Samo Gudo

**Affiliations:** 1 National Institute of Health, Ministry of Health, Field Epidemiology and Laboratory Training Programme-Mozambique, PO Box 264, Av Eduardo Mondlane 1008, Ministry of Health Main Building, 2nd floor, Maputo, Mozambique; 2 Department of Microbiology, Faculty of Medicine, Eduardo Mondlane University, PO Box 257, Av. Salvador Allende 702, Maputo, Mozambique

**Keywords:** Malaria, Pregnant woman, IPTp, Insecticide-treated mosquito nets, Mozambique

## Abstract

**Background:**

Malaria during pregnancy is associated with poor maternal and pregnancy outcome and the World Health Organization recommends the administration of intermittent preventive treatment in pregnancy (IPTp) with sulfadoxine-pyrimethamine (SP) and distribution of insecticide-treated mosquito nets (ITNs) to all pregnant women attending antenatal care (ANC) services. This study was conducted with the aim to assess the uptake of IPTp and ITNs in pregnant women attending ANC services and correlate with ANC attendance and frequency of stock-outs in 22 health facilities Mozambique.

**Methods:**

A cross-sectional study was conducted between July and December 2011 in 22 health units in 11 districts situated in 11 provinces in Mozambique. Two health facilities were selected per district (one urban and one rural). Data were collected by reviewing logbooks of antenatal consultations as well as from monthly district reports.

**Results:**

During the period under investigation, a total of 23,524 pregnant women attended their 1st antenatal care visits, of which 12,775 (54.3%) and 7581 (32.2%) received one and two doses of IPTp, respectively. In regard to ITNs, a total of 16,436 (69.9%) pregnant women received ITNs. Uptake of IPTp and ITNs by pregnant women at ANC services was higher in southern Mozambique and lower in districts situated in the northern part of the country. Stock-outs of SP and ITNs were reported in 50.0% (11/22) and 54.5% (12/22) of the health facilities, respectively. Coverage of IPTp and ITN in health facilities with stock-outs of SP and ITNs was much lower as compared to health facilities with no stock-outs.

**Conclusions:**

Altogether, data from this study shows that coverage of the 2nd dose of IPTp, as well as ITNs, was low in pregnant women attending ANC services in Mozambique. In addition, this data also shows that stock-outs of SP and ITNs were frequent and led to lower coverage of IPTp and ITN, representing a serious barrier for the accomplishment of targets. In conclusion, this study recommends that efforts should be made to improve the supply chains of SP and ITNs.

## Background

Malaria in pregnancy remains a major public health problem in endemic countries, mostly in sub-Saharan Africa, the region that carries the heaviest burden. According to the 2015 Global Malaria Report, 87.9% of all malaria cases and 90.2% of all malaria deaths reported worldwide in 2015 occurred in sub-Saharan Africa [1]. Acquisition of malaria during pregnancy is associated with poor outcomes, which include maternal anemia, low birth weight, and higher risk maternal and child mortalities [2–4]. Worldwide it is estimated that 10,000 of all maternal deaths and 200,000 of all neonatal deaths are attributable to malaria [5].

In countries with moderate to high transmission, the World Health Organization (WHO) recommends intermittent preventive treatment in pregnancy (IPTp) with sulfadoxine-pyrimethamine (SP), together with the distribution of insecticide treated nets (ITNs) for all pregnant women attending for antenatal care (ANC) services [2]. As per the WHO recommendation, pregnant women should receive SP at each ANC visit until delivery, starting in the second trimester of pregnancy, with subsequent doses provided at least 1 month apart [2, 6]. In Mozambique, SP is administered under directly observed therapy (DOT) as per WHO recommendation [2].

Although the majority of countries in sub-Saharan Africa have adopted WHO guidelines for malaria prevention in pregnancy [7], results of studies conducted in several countries in the region demonstrate that the coverage of IPTp and ITNs in pregnant women is still low [8, 9]. Stock-outs of SP and ITNs, a lack of awareness of health care workers at ANC services [10–12], inconsistencies of national policies [7], among other factors were identified as barriers for implementation of these intervention in several countries.

In regards to Mozambique, available evidence demonstrates that malaria is a leading cause of maternal mortality, being responsible for up to 26.9% of deaths in southern Mozambique [13–15]. The National Malaria Programme adopted the WHO guideline for malaria prevention in pregnancy in 2006, which since then has been offered free of charge in the public sector in Mozambique [16]. Two studies conducted in the southern region provided convincing evidence of the program’s cost-effectiveness in reducing maternal and neonatal mortality in the country [17, 18]. However, data from repetitive nationwide household surveys demonstrate the coverage of both IPTp and ITNs is still low [19–22], and a meta-analysis conducted by van Eijk et al. in 2013 showed that the coverage in Mozambique is lower when compared with several countries in the region [9].

Stock-out of SP and ITNs has consistently been considered a major cause for low coverage in pregnant women in other countries [11, 12]; however, no study has yet been conducted to understand the possible role of stock-outs on coverage of IPTp and ITNs in pregnant women in Mozambique. Indeed, data on the determinants of low coverage of these interventions are limited in Mozambique, and the few studies conducted in the country focused their research on knowledge and acceptability of this intervention among pregnant women [23]. Moreover, current estimates on coverage in Mozambique are based mostly on data from household surveys alone. In this regard, this is the first facility-level survey to determine the coverage of IPTp and ITNs and investigate the influence of stock-outs of SP and ITNs on the coverage of IPTp and ITNs at ANC services in 22 health facilities across 11 districts in 11 provinces in Mozambique. According to what is known this represents the first attempt to correlate coverage with stock-out of SP and ITNs.

Results of this study are of utmost importance for the definition of interventions for improving prevention of malaria in pregnancy in Mozambique.

## Methods

### Study design and study setting

A retrospective study was conducted in 11 districts in 11 provinces throughout the country between July and December in 2011. During the study period, data about ANC visits corresponding to January and December of this same year was collected. Within each district, two health facilities were selected, one situated in a rural area and the second situated in an urban setting, which in total comprised 22 health facilities. At each setting (rural or urban/sub urban), the health facilities with highest demand at ANC services were selected.

Mozambique is situated on the southeastern coast of Africa, with a total area of 801,590 square km and over 2500 km of coastline. The climate is typically tropical with two distinct seasons, the rainy season from November to April and the dry season from May to October. The relative humidity is high and ranges between 70 and 80%. The average annual precipitation is estimated to be 600 mm, and varies between 500 and 900 mm. The total population of the country is estimated to be 27 million, of which approximately 70% lives in rural areas [24]. Smallholder agriculture and fishing represents the main source of income [25, 26]. The country is administratively divided into 3 regions (north, center, and south) and has 11 provinces and 128 districts. Mozambique is highly vulnerable to natural disasters such as droughts, cyclones, and floods that often contribute to increased transmission of malaria.

### IPTp and ITN supply chain in Mozambique

SP-IPTp and ITN are supplied to the health facilities separately through independent supply chain mechanisms. SP-IPTp is supplied through the national supply chain mechanism that is used for routine distribution of most of the medical products and equipment at public sector which is coordinated by a central warehouse for medical products based in the capital of the country. From this central warehouse, most medical products including SP-IPTp are distributed at every 3 months to the provincial medical warehouse at each of the 11 provincial capitals. At every month, each medical product or supply is distributed from the provincial medical warehouse, to each district medical warehouse which subsequently distributes to each health facilities on a monthly basis. The monthly request of SP—IPTp from the health facility to the district medical warehouse is under responsibility of the maternal and child health nurse who is in charge of ANC services at health facility. The request is done using the following formula: (average of weekly consumption × 9—existing stock).

The supply of ITNs is under the responsibility of the country’s National Malaria Control Programme. Distribution of nets to each health facility follows a similar supply chain mechanism, but at each distribution hub (national, provincial and district warehouse), the process is coordinated by the national, provincial or district malaria focal person. At health facility, the monthly request is also done by the maternal and child health nurse who is in charge of ANC services, based on the calculation of the average number of ANC consultations each month.

### Data collection

At each health facility, data on the number of pregnant woman attending ANC services, number of ANC visits, number of 1st and 2nd doses of SP administered, and number of ITNs offered to pregnant woman were collected by reviewing the logbooks at ANC services, as well as from the monthly district statistics and reports on maternal and child health. Data was collected by trained health workers using a standard questionnaire.

### Data analysis

Data was double-entered by two different data clerks into a database developed using Epi Info v 3.5.4 (2008). Both entries were then matched for correction of errors during data entry. Coverage of IPTp and ITNs was defined as the proportion of pregnant women attending ANC services who had received SP or ITNs. Analysis was performed using the SPSS statistical software, version 17 (IBM Corp., Armonk, NY, USA), to calculate the frequencies, proportions, and bi-variate analysis.

## Results

Between July and December 2011, a total of 23,524 pregnant women attended ANC service for their 1st visit across the 22 health facilities; of which 12,775 (54.3%) received one dose of IPTp, and 16,436 (69.9%) received ITNs (Table 1). Remarkably, only 7581 (32.2%) of them received two doses of IPTp, which is almost half when compared with those who received only one dose of IPTp (Table 1).

**Table 1:** Proportion of pregnant women receiving 1st and 2nd IPTp and ITNs in 22 health facilities across 11 districts, stratified by districts in Mozambique, 2011

District	PW attending 1st ANC (n)	PW receiving 1st dose of IPTp (n, %)	PW receiving 2nd dose of IPTp (n, %)	PW receiving ITNs (n, %)
Northern	10,342	5013 (48.5)	3222 (31.2)	5117 (49.5)
Ngauma	2989	1142 (38.2)	730 (24.4)	1359 (45.5)
Murrupula	3825	2310 (60.4)	1438 (37.6)	1597 (41.8)
Montepuez	3528	1561 (44.2)	1054 (29.9)	2161(61.3)
Central	9411	5418 (57.6)	2314 (24.6)	7921 (84.2)
Changara	1023	712 (69.6)	435 (42.5)	839 (82.0)
Caia	938	616 (65.7)	491 (52.3)	542 (57.8)
Manica	2359	1916 (81.2)	1145 (48.5)	2359 (100)
Alto-Molocue	5091	2174 (42.7)	243 (4.8)	4181 (82.1)
Southern	3771	2344 (62.2)	2045 (54.2)	3384 (89.7)
Zavala	887	373 (42.1)	451 (50.8)	887 (100)
Chibuto	1879	1231 (65.5)	1017 (54.1)	1529 (81.4)
Magude	841	620 (73.7)	479 (57.0)	843 (100)
Urban district no 1	164	120 (73.2)	98 (59.8)	125 (76.2)
Total	23,524	12,775 (54.3)	7581 (32.2)	16,422 (69.8)

### Coverage of IPTp and ITN is heterogeneous in different geographical regions in Mozambique

The coverage of 1st and 2nd doses of IPTp in pregnant women varied in different geographical regions (Table 1), and the southern region of Mozambique presented a higher coverage for both indicators as shown in Table 1. The coverage of ITNs in pregnant women also varied by geographical region with a higher coverage noted in the southern region. Of note, a 100% coverage rate of ITNs in pregnant women was achieved in Manica and Magude districts in Manica and Maputo provinces, respectively.

For a better understanding of administration of preventive measures of malaria in pregnancy at ANC, the number and proportion of ANC visits in which 2nd doses of SP as well as ITNs were given to pregnant women was calculated, as shown in Table 2. Between January and December 2011, a total of 94,044 ANC visits were conducted and recorded, but in only 20.5% (19,300/94,044) of them, the minimum of two doses of IPTp were administered, where as in 85.6% (80,469/94,044) of visits, ITNs were offered to the women. These indicators also varied in different geographical regions, as we noted that the higher proportion of ANC visits in which IPTp were offered to the pregnant women was in the southern region and the lowest was reported in northern region. Of remark, data from Table 1 shows that the lowest rate coverage of IPTp was reported in Montepuez district in Cabo Delgado province situated in northern Mozambique (10.9%; 874/7986), and the highest coverage rates were reported in Zavala district in Inhambane province and Magude district in Maputo province, both in southern Mozambique, reported [(36.5% (1972/5399) in Zavala district and 36.5% (826/2265) in Magude district, respectively)].

**Table 2:** Number of ANC visits in which two doses of IPTp and ITNs were provided to pregnant women in 22 health facilities across 11 districts, stratified by districts in Mozambique in 2011

District	Number of 1st ANC visits (n)	Coverage of 2nd dose of IPTp at ANC (n, %)	Coverage of ITNs at ANC (n, %)
Northern	24,996	3894 (15.6)	20,608 (82.4)
Ngaúma	5415	841 (15.5)	4961 (91.6)
Murrupula	11,595	2179 (18.8)	7661 (66.0)
Montepuez	7986	874 (10.9)	7986 (100)
Cental	50,599	10,987 (21.7)	41,611 (82.2)
Changara	7049	1183 (16.8)	4121 (58.5)
Caia	6049	1516 (25.0)	5749 (95.0)
Manica	14,703	1960 (13.3)	14,554 (98.9)
Alto-Molócue	22,798	6328 (27.7)	17,187 (75.4)
Southern	18,449	4419 (24.0)	18,250 (98.9)
Zavala	5399	1972 (36.5)	5399 (100)
Chibuto	10,574	1586 (14.9)	10,574 (100)
Magude	2265	826 (36.5)	2066 (91.2)
Urban district no 1	211	35 (16.6)	211 (100)
Total	94,044	19,300 (20.5)	80,469 (85.6)

The proportion of ANC visits in which ITNs were offered to pregnant women also varied by geographical region (Table 2). Changara district in Tete province situated in the central part of the country reported the lowest coverage of ITNs in pregnant woman, being offered in 58.5% (4121/7049) of visits. On the other hand, higher coverage of ITNs was reported in Montepuez district in Cabo Delgado in northern Mozambique as well as in Zavala in Inhambane, Chibuto in Gaza, and Urban district no 1 in Maputo City, all in southern Mozambique, which reported coverage of 100% (Table 2).

### Influence of stock-outs of SP and ITN associated in lower coverage of IPTp and ITNs at ANC consultations

This study also investigated the influence of stock-outs of SP and ITNs on the coverage of IPTp and ITNs. Table 3 shows that 6800 (29.2%) out of 23,288 pregnant women attended their 1st ANC visit in health facilities with stock-out of SP in the period under investigation, and the coverage of the 1st dose of IPTp in health facilities with stocks out of SP was half of that reported in health facilities with no stock-outs of SP [coverage of 34.1% (2320/6800) in health facilities with stock-outs versus 62.5% (10,288/16,488) in health facilities with no stock-outs of SP]. With regards to the 2nd dose of IPTp, the coverage in health facilities with stock-outs of SP was as low as 3.7% (251/6800), while in health facilities with no report of stock-outs of SP, the coverage was 43.6% (7172/16,448).

**Table 3:** Frequency of pregnant women attending ANC visits who received 1st and 2nd dose of IPTp and ITNs stratified by presence or absence of stock-out and type of health facility

Classification of health facility	PW attending 1st ANC (n, %)	Coverage of 1st dose of IPTp at ANC (n, %)	Coverage of 2nd dose of IPTp at ANC (n, %)	Coverage of ITNs at ANC (n, %)
Reporting of stock out of ITP				
With report of stock out of ITP	6800 (29.2)	2320 (34.1)	251 (3.7)	–
Without report stock out ITP	16,448 (70.8)	10,288 (62.5)	7172 (43.6)	–
Reporting of stock out of ITN				
With report of stock out of ITN	13,211 (58.3)	–	–	5969 (45.2)
Without report stock out ITN	9466 (41.7)	–	–	10,111 (106.8)
Location of health facility				
Urban	10,731 (45.5%)	5928 (55.2)	3322 (30.9)	8577 (79.9)
Rural	12,829 (54.5%)	6847 (53.4)	4183 (32.6)	7217 (56.2)
Total	23,524	12,775 (54.3)	7581 (32.2)	16,422 (69.8)

Table 3 also shows that 13,211 (58.3%) out of 22,677 pregnant women attended their 1st ANC visit in health facilities with stock-outs of ITNs in the period under investigation, and the coverage of ITNs in health facilities with stock-outs of ITNs was 45.2% (5969/13,211); while in health facilities with no stock-outs of ITNs, the coverage was 106.8% (10,111/9466).

Coverage of IPTp and ITNs in health facilities situated in urban and rural areas was also compared. Table 3 shows that coverage of IPTp was similar in both types of zones, but distribution of ITNs was lower in health facilities situated in rural areas as compared to those in urban areas (56.2%, or 7217/12,829, in rural areas versus 79.9%, or 8577/10,731, in urban areas).

**Fig. 1:**
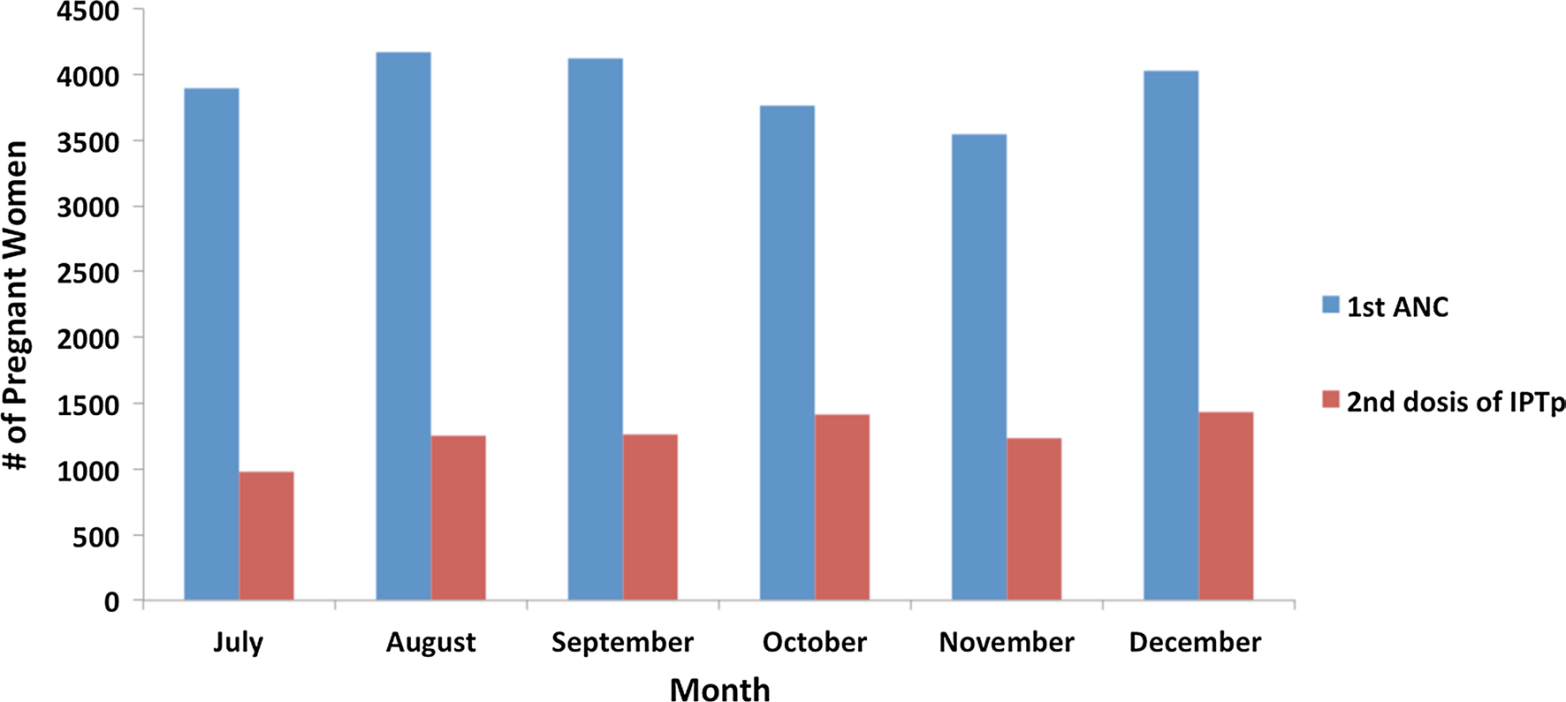
Monthly variation of pregnant woman attended at ANC and those who received IPTp, in 22 health facilities visited from July to December 2011 (Source: Record books of pregnant women consultations at the 22 health facilities visited)

**Fig. 2:**
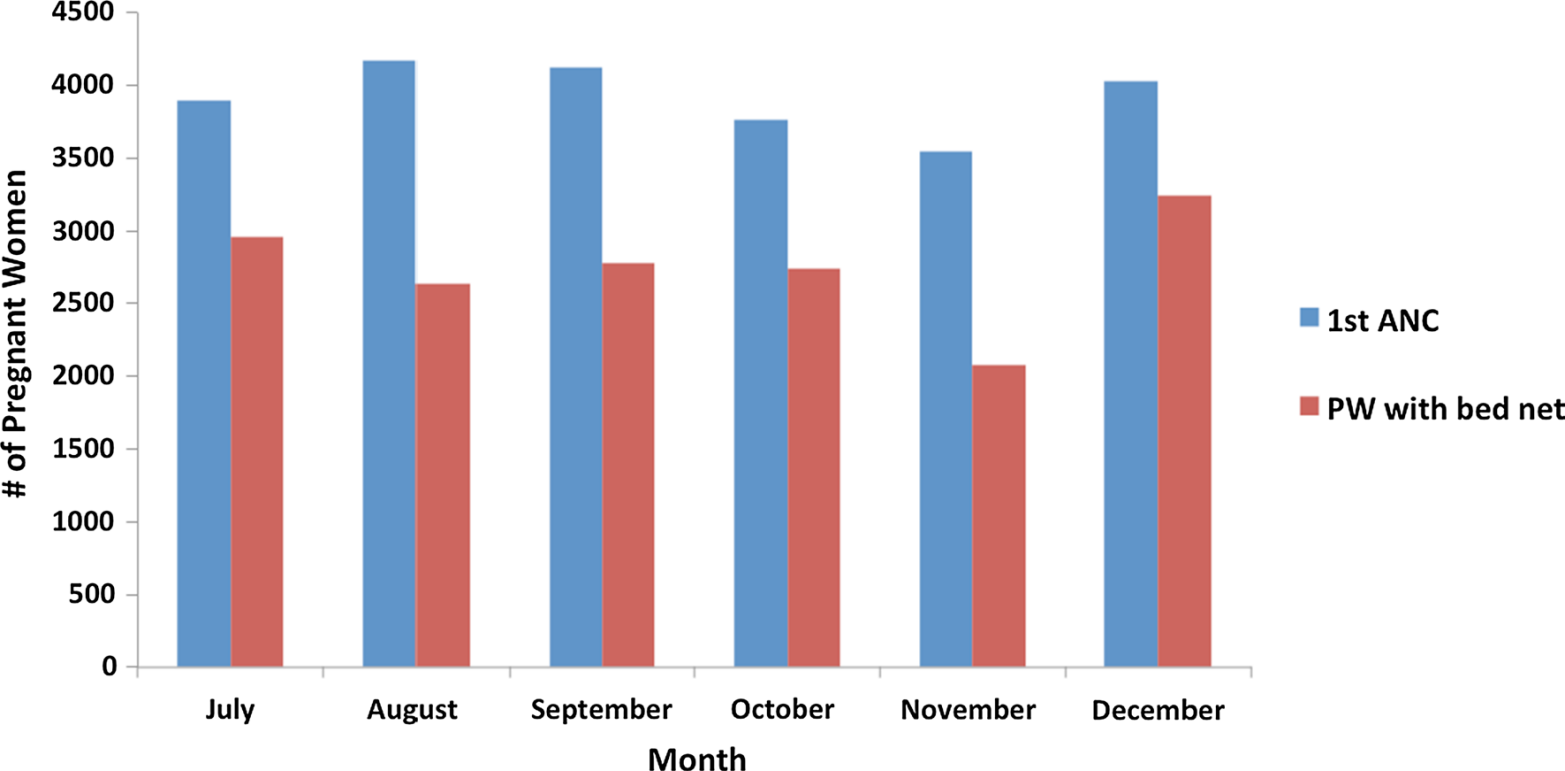
Monthly variation of pregnant woman attended at ANC and those who received mosquito nets, in 22 health facilities visited from July to December 2011 (Source: Record books of pregnant women consultations at the 22 health facilities visited)

The proportion of pregnant women receiving IPTp and TNs did not vary significantly by month as shown in Figs. 1 and 2. Data from Fig. 2 also showed that the lowest coverage of ITN was reported in November (58.5%, or 2077/3547) and the highest in December (80.5%, or 3244/3891).

## Discussion

Prevention of malaria during pregnancy consisting of administration of SP as well as ITNs to women during their ANC visits is effective in reducing the risk of poor outcomes in pregnant women and was adopted as policy in 2006 in Mozambique [27–29]. However, there is limited data on the determinants of the sub-optimal coverage reported in repetitive surveys, in particular the role of service delivery; no survey has yet been conducted using facility-level data. This is particularly relevant in Mozambique where maternal and neonatal mortalities and the burden of malaria are among the highest in the Southern Region of Africa [1, 30, 31].

This study investigated the coverage of these interventions among pregnant women attending ANC services in 22 health facilities in 11 districts in Mozambique in 2011. Data from this study shows that in all health facilities the proportion of pregnant women receiving the minimum of two doses of IPTp at ANC services was quite low (32.2%). Although data from several neighboring countries, as well as the worldwide average, have been higher than Mozambique’s [11, 32–34], data from WHO shows that in sub-Saharan Africa, the proportion of pregnant women attending ANC services who received a 2nd dose of IPTp ranged from 30 to 57% in 2011 [35].

The reason for the low adherence to WHO guidelines at ANC services is not well-known in Mozambique, but findings from other countries suggest that this may be attributed to several factors such as the stock-outs, a low level of awareness among health care workers, inconsistent policies, a lack of training and supervision, and late initiation of ANC visits [10–12].

This study investigated for the first time the possible role of stock-out in the coverage of ITN and IPTp. Data from this study show that stock-outs of SP and ITN were reported in 29.2 and 58.3% of the health facilities, respectively, suggesting that interventions are urgently needed to improve supply chains for SP and ITNs countrywide. On the other hand, coverage of both SP and IPTp was lower in health facilities with stock-out as compared to those with no stock-out. These findings provide convincing evidence that stock-outs might represent an important barrier for the effective delivery of this intervention as shown in many other countries [10–12]. Remarkable, data from this study found that coverage of ITN in health facilities without stock-out of ITN was above 100%. In sub-Saharan Africa, including Mozambique, over-coverage is seen frequently for several health indicators [36, 37]. Possible causes to explain over percentage includes errors in reporting routine data and tendency to over-reporting, as previously suggested [36]. Indeed, several studies have shown that nurses are overwhelmed of work and do not have enough time for documenting [38].

Previous studies in other countries showed that irregular training of health care workers, as well as irregular supervision, represents barrier for the uptake of interventions to prevent malaria during pregnancy [10, 12]. Although the possible role of training and supervision was not investigated in this study, there is a belief that lack of regular training and supervision contributes to a lower level of uptake of both interventions in Mozambique. This is corroborated also by the fact that only half of pregnant women attending their 1st ANC visit received SP in the absence of stock-outs of SP, strongly suggesting that health care workers’ practices represent an important barrier for the implementation of this intervention. This highlights the urgent need to implement strategies for changing health care workers’ practices, such as refresher trainings, intensification of site visits, and institutionalization of tools for continuous monitoring of the coverage of these interventions. Moreover, in 2013 the WHO highlighted that one of the reasons for the slow scale-up of IPTp among pregnant women is attributed to confusion and a lack of understanding among clinicians about the recommendation for the administration of SP during pregnancy [2]. Similar findings were reported by Hill et al. [12].

In this study, results on coverage of both 1st and 2nd dose of SP were only slightly higher than those reported at community levels during the Demographic and Health Survey (DHS) conducted in 2011 [20], yet similar to those reported in the more recent Malaria Indicator Survey (MIS) conducted in 2015 [21], which suggest that coverage is increasing, although slowly.

Data from the global Multiple Indicator Cluster Survey showed that attendance of at least one ANC visit in Mozambique have increased from 85% in 2003 to 91% in 2011 and 93% in 2015 [20–22]. Moreover, data from the MIS conducted in 2007 showed that 84% of pregnant women attend at least two ANC visits during pregnancy [19], and data from the most recent MIS showed that 55% of pregnant women actually attend at least four ANC visits. This suggests that the steep decline in the coverage of SP between the 1st to the 2nd ANC visits should not be entirely attributed to the rate of return to the second visit, which reinforces the hypothesis that health care workers’ practices represents a main barrier. This is also corroborated by findings from studies conducted in other countries that similarly showed that, while attendance of ANC services increased significantly, coverage of IPTp still remains low [12, 32, 36].

Although no data on gestational age during ANC visits was obtained, there is a belief that the late initiation of ANC services by pregnant women represented an additional barrier to ensuring adequate coverage of IPTp. It is well-known that late attendance is a common finding in sub-Saharan African countries [37–39], and, as a consequence, more than one dose of SP is no longer a realistic target. Indeed studies conducted in the region have demonstrated that delayed visits to antenatal services represented a major obstacle for the prevention of malaria during pregnancy [10, 12, 35]. This highlights that combined efforts are needed to ensure that women attend ANC services sooner in pregnancy and return for follow-up visits. According to WHO guidelines, the percentage of pregnant women receiving the second dose of IPTp is unlikely to reach 100% because some pregnant women do not ever return for the second visit, however it must be above 80% [40].

The coverage of ITNs (69.8%) found in this survey is much higher than reported in the 2011 DHS and 2015 MIS, which reported that at a community level only 20% and 47.9% of pregnant women slept under ITNs the previous night, respectively [20, 21]. However, results of this study are similar to those reported among pregnant women during a household survey in Zambézia in 2014 [41]. This highlights that, although coverage is increasing across the country, it is still below the target. This is still concerning as it demonstrates that there is a significant number of pregnant women at high risk of developing malaria, leading to poor maternal and child health outcomes. Data from this study suggests that campaigns should particularly prioritize pregnant women in order to supplement the coverage, as two recent studies conducted in Mozambique demonstrated the mass campaigns distribution could achieve very high ITN coverage of households [42, 43].

As previously mentioned, there were geographical differences in the coverage of IPTp and ITNs among pregnant women attending ANC services. For both indicators, the coverage was higher in the southern region and lower in the northern part of the country. This may reflect differences in awareness, knowledge, and practices of health care workers in various parts of the countries. This highlights that more interventions to improve distribution of IPTp and ITNs should be intensified in the northern region, specifically.

This study has a few limitations that should be acknowledged, such as the quality of routine data that resulted in over-coverage of ITN in health facilities without stock-out, as well as the lack of prior studies on the coverage of ITN and SP-IPTp using facility based data that could be used for comparison.

## Conclusions

Altogether, data from this study show that coverage of the 2nd dose of IPTp, as well as ITNs, is low in PW attending ANC services in Mozambique. This study also found that stock-outs of SP and ITNs were frequent and led to a lower coverage of IPTp and ITNs, representing a serious barrier for the accomplishment of targets. Moreover, the worst rates of coverage were observed in northern part of the country, implying the need for more geographically-focused programs. Interventions to revert this scenario should include (i) increasing the awareness of health care workers through massive refresher trainings accompanied by intensification of site visits; (ii) improvement of supply chains to reduce the frequency and duration of stock-outs of SP and ITNs; (iii) community mobilization to increase the attendance of pregnant women for follow-up ANC visits; and (iv) institutionalization of performance indicators at ANC services for real time monitoring of the coverage of these interventions allowing more immediate changes to be made.

## Abbreviations

ANC: antenatal care
DHS: demographic and health survey
DOT: directly observed therapy
ITNs: insecticide-treated mosquito nets
IPTp: intermittent preventive treatment in pregnancy
MIS: malaria indicator survey
PW: pregnant women
SP: sulfadoxine-pyrimethamine
WHO: World Health Organization
